# Current role of portable MRI in diagnosis of acute neurological conditions

**DOI:** 10.3389/fneur.2023.1255858

**Published:** 2023-09-29

**Authors:** Arya Shoghli, Daniel Chow, Edward Kuoy, Vahid Yaghmai

**Affiliations:** Department of Radiological Sciences, School of Medicine, University of California, Irvine, Irvine, CA, United States

**Keywords:** MRI, portable MRI, neuroimaging, stroke, ICH, point-of-care (POC)

## Abstract

Neuroimaging is an inevitable component of the assessment of neurological emergencies. Magnetic resonance imaging (MRI) is the preferred imaging modality for detecting neurological pathologies and provides higher sensitivity than other modalities. However, difficulties such as intra-hospital transport, long exam times, and availability in strict access-controlled suites limit its utility in emergency departments and intensive care units (ICUs). The evolution of novel imaging technologies over the past decades has led to the development of portable MRI (pMRI) machines that can be deployed at point-of-care. This article reviews pMRI technologies and their clinical implications in acute neurological conditions. Benefits of pMRI include timely and accurate detection of major acute neurological pathologies such as stroke and intracranial hemorrhage. Additionally, pMRI can be potentially used to monitor the progression of neurological complications by facilitating serial measurements at the bedside.

## Introduction

Neurological disorders are the second leading cause of death with 9.0 million deaths per year globally and the leading cause of disability with disability-adjusted life-years (DALYs) of 276 million (1, 2). Almost half of the burden of neurological diseases takes place in low-income and middle-income countries (3). Magnetic resonance imaging (MRI) is the preferred imaging modality for neurological disorders. However, MRI scanners are costly, immobile, and lack timely availability. This potentially delays diagnosis and management. The development of low-cost portable MRI (pMRI) devices has enabled point-of-care (POC) neuroimaging. The pMRI device was first cleared by the U.S. Food and Drug Administration in August 2020 (4). This article will briefly introduce pMRI technology. Then we review the current role of pMRI in acute neurological and critical care settings. Finally, we conclude with a discussion on the current scope and ongoing opportunities.

## Neuroimaging modalities

Neuroimaging is a critical step for timely triage, diagnosis, and decision-making for treatment of patients with clinically suspected acute conditions such as stroke (4–6). Non-contrast computed tomography (CT) has historically been a cost-effective imaging modality for the initial evaluation of neurologic patients (7, 8). However, studies have increasingly demonstrated that multimodal MRI has higher sensitivity for detecting many acute neurological conditions. Improved soft tissue contrast, lack of radiation exposure, and detection of small infarcts are the reasons for MRI's superiority over CT (9, 10).

## Portable MRI

**Figure 1:**
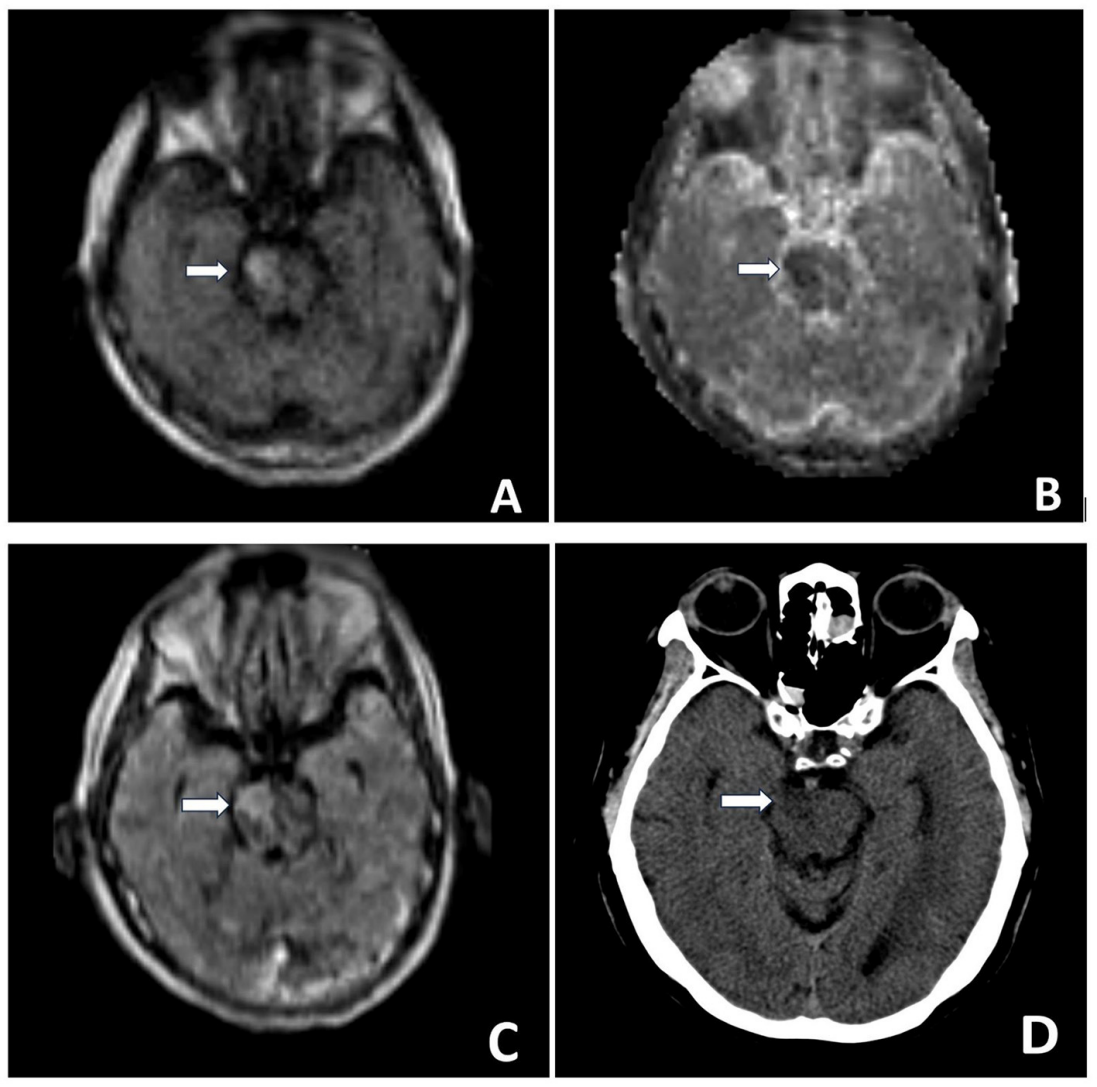
Brain MR POC MRI DWI sequence **(A)**, ADC sequence **(B)** demonstrate right ventral pontine restricted diffusion. T2/FLAIR sequence **(C)** demonstrates right ventral pontine hyper-intense signal. Head CT performed the same day **(D)**: Hypo-attenuation at the site of right pontine infarction, but at times difficult to confidently diagnose posterior fossa strokes on CT given artifacts in the area. MRI exam helps verify diagnosis. MR Images were obtained using the Swoop Hyperfine Portable MR Imaging System (64 mT) at the University of California, Irvine Medical Center.

Conventional MRI with high-strength magnetic field (1.5–3 T) systems are large, immobile, costly, and require special infrastructure, rigid safety precautions, and highly trained technicians (4, 11, 12). Usage of the pMRI with a considerably lower strength (64 mT) allows for 3D, whole brain Diffusion-weighted imaging (DWI), Fluid-attenuated inversion recovery (FLAIR), T2-weighted (T2W), and T1-weighted (T1W) imaging (Figure 1). DWI is recognized as a superior method for detection of acute infarcts by providing the most relevant contrast. FLAIR, T1W, and T2W are highly sensitive in detection of subacute and chronic ischemic brain infarcts and observation of response to treatment (9). Some of the advantages of pMRI include:

(1) POC neuroimaging in emergency settings (13–19) or for critically ill patients in intensive care units (ICU) (11, 20, 21); (2) Reduced risk of transmission of infections during pandemics like coronavirus disease 2019 (COVID-19) (12, 22, 23); (3) Logistical access for brain MR imaging (24–26) in the ICU or in remote low- and middle-income regions (9, 27–29); (4) Fewer safety concerns regarding intra-hospital transport due to required restrictions for conventional clinical MRI (30–33); (5) Cost-effectiveness (34–37); (6) Reduced hospital length of stay (LOS) (11, 15, 21); (7) No risk of thermal burns and ferrous projectiles; (8) Detection of intracranial pathologies similar to that observed on conventional MRI by offering different pulse sequences (4, 14, 38).

## Role of pMRI in acute neurological conditions

### Strategies for pMRI imaging in the emergency department

Lang et al. have proposed dividing the usage of pMRI imaging in ED into three groups based on their field strength and portability (39). The first group are “easy-to-site suite” scanners using a standard superconducting solenoid magnet architecture; Examples are High-field (3T) (40, 41) and Mid-field (between 0.5 T and 1.0 T) head-only MRI scanners (42–44). The second group would be truly portable scanners that would operate at a low field (50 mT to 200 mT), need unconventional electromagnetic interference (EMI) mitigation, and must operate with substantially reduced electrical power and without water cooling or cryogenics. The 64 mT Hyperfine Swoop scanner (Guilford, CT, USA), the first FDA-cleared clinical pMRI scanner (12, 45), and the 80 mT “Halbach-bulb” rare-earth magnet configuration (38, 46, 47) are some of the advances in this class. The ultra-low-field shield-free pMRI using a Samarium-cobalt magnet enables the cancellation of EMI and elimination of the traditional radiofrequency shielding cage. This scanner promisingly met the clinical needs to diagnose brain tumors and stroke (48). The third group are “hand-held” level MRI devices, likely with greatly reduced imaging capabilities, but inexpensive and small enough to be considered MR detectors or monitoring devices more than diagnostic imaging devices. An example of this is a 7 kg device “MR Cap” (49). This type of instrument could warn of early changes and impending events by continuously imaging the brain in ED or ICU, particularly in patients that are difficult for clinical evaluation and examination (e.g. sedated patients).

### Stroke

Stroke is the second leading cause of mortality and morbidity worldwide and affects 15 million people each year. Neuroimaging plays a significant role in both the diagnosis and treatment of stroke within the golden time (first 3–4.5 h after the event). The availability of open-bore systems has enabled better temporal access than fixed CT or conventional MRI and resulted in timely therapeutic intervention and reduced door-to-needle time (9). DWI remains the gold standard sequence imaging for acute stroke and is available on low-field and very low-field/ultra-low-field MR scanners (29). In 2020, Cahn et al. suggested the clinical utility of POC MRI for evaluation of stroke patients. Using the 64 mT scanner (50), they examined acute stroke patients and were able to produce 3D clinical quality images by performing DWI sequences and suggested it as a safe and viable tool in complex clinical care environments (17). Zubair et al. (13) and Hovis et al. (15) in 2021 reported using 64 mT pMRI for evaluation of patients with suspected stroke. The implementation of pMRI resulted in faster work-up, decreased hospital stays, and yielded reliable results compared to non-contrast CT scan and conventional MRI. In 2022, Yuen et al. utilized 64 mT pMRI as first-line diagnostic imaging for the detection of ischemic stroke. POC MRI was able to capture lesions as small as 4 mm. Infarcts were detected as hyper-intense regions on T2-W, FLAIR, and DWI sequences with a sensitivity of 98, 100, and 86%, respectively. Stroke volume measurements were consistent between low-field pMRI and conventional high-field MRI studies. Significant correlations were seen between low-field pMRI stroke volumes and stroke severity, and consequently functional outcome at discharge. They validated the clinical use of low-field pMRI as a novel imaging solution for patients with ischemic stroke, especially in resource-limited environments (18).

#### Mobile imaging of stroke

The mobile stroke unit was first proposed hypothetically by Fassbender et al. for evaluation and diagnosis of stroke considering timely accessibility (51) and was first introduced by Parker et al. in 2015 (52). It is a combination of emergency medical services, telemedicine, and a portable CT scanner (53). Low-field pMRI could potentially provide POC service in this setting. Portable low-field scanners do not require additional shielding and have a similar footprint as portable CT scanners. Therefore, imaging of stroke patients even in remote regions or resource-limited geographies would be possible (9). Deoni et al. assessed the feasibility of a cargo van equipped with 64 mT low-field pMRI. The data collected by the mobile system showed no significant differences regarding geometric distortion, signal-to-noise ratio, or tissue segmentation compared to laboratory settings. Their results illustrated a promising approach that allows for neuroimaging of participants at home, school, etc. (54). However, one significant challenge in mobile unit implementation is motion artifact.

### Intracerebral hemorrhage and midline shift

In 2020, Shah et al. analyzed the ICH detection capacity of a bedside POC MRI scanner. Prospectively, using a 64 mT scanner, a pathologic lesion was identified on every exam with 100% sensitivity by T2-W and FLAIR sequences. They suggested that low-field, POC MRI may be used to detect hemorrhagic strokes at the bedside (19). In 2021 Mazurek et al. evaluated the use of low-field MRI (64 mT) for assessing ICH in comparison with conventional 1.5/3 T MRI or non-contrast CT scan. PMRI could detect ICH with 80.4% sensitivity and 96.6% specificity. They found that hematoma volume measurements derived from pMRI exams correlated with impaired cognitive status and worse functional capacity at discharge. They proposed the Low-field pMRI as a useful technology in resource-limited settings (16). With the same methodology, Sheth et al. assessed mass effects and the resulting MLS in brain-injured patients using pMRI. There was significant agreement between pMRI and conventional MRI or CT. Low-field pMRI identified MLS with a sensitivity of 93% and specificity of 96% and could predict poor clinical outcomes at discharge. They regarded bedside pMRI as valuable for detecting mass effects (14).

### Critically ill patients

In patients admitted to the ICU, transportation to an MRI suite may place the patient at numerous risks, including compromise of venous or arterial access, endotracheal tube displacement, hypoxia, hypotension, increased intracranial pressure, and cardiac arrest. In patients with contagious infections such as COVID-19 other concerns such as exposure to a larger patient population and hospital staff and the need for decontamination of radiology suites are logistical barriers (12, 22). On the other hand, substantial delays between ordering and obtaining an MRI may further increase patients' hospital length of stay and subsequent total hospitalization costs. Importantly, limited access to MRI hinders timely and targeted therapeutic decision-making in this patient population (4, 11, 21).

Low-field pMRI (64 mT) produces less than 5 Gauss beyond the 5-foot (1.52 meter) safety zone of the magnet's center, which is significantly lower than conventional MRI. It can be feasibly employed in the presence of all ICU equipment during scanning without adverse events (26). Additionally, POC MRI may improve the functionality of existing fixed MRI machines by removing complicated and time-sensitive critically ill patients from the scheduled waiting list (11, 20, 21). In 2020–21, Turpin et al. (22) and Mazurek et al. (23) reported their experience of imaging critically ill COVID-19 patients with a self-shielding, 64 mT pMRI. The use of pMRI was safe, and feasible and led to changes in clinical management based on imaging results. Also, the diagnostic MRI quality was graded to be adequate (85%) with FLAIR sequences demonstrating the highest quality, and DWI scoring the lowest (22). Similarly, Sheth et al. assessed the utility of pMRI in complex clinical settings such as neuroscience labs and COVID-19 ICUs and reached similar results (12). However, Kuoy et al. obtained a diagnostic rate of 72% and mentioned that motion artifact, habitus, and lower field-strength may necessitate higher field-strength MRI subsequently. They also found that the turnaround time was reduced for patients in the ICU (5.3 vs. 11.7 h for fixed scanner) but not for patients in the ED (3.4 vs. 3.7 h for fixed scanner), suggesting that ease of transport and proximity to the fixed scanner may affect turnaround times (21). Regarding the time-dependent nature of critically ill patients and particularly those resuscitated from cardiac arrest, Beekman et al. demonstrated that pMRI could successfully identify patients with hypoxic-ischemic brain injury (HIBI). Accurate outcome prediction is required to prevent withdrawal of life-sustaining therapy in comatose survivors with good functional outcomes; this is possible through serial MR imaging and quantitative analyses using FLAIR intensity (11). The same applies to patients on extracorporeal membrane oxygenation (ECMO) as both logistic and diagnostic advantages of POC MRI led to early detection and timely intervention in acute brain injuries and could improve their outcomes (20).

## Other applications

The application of pMRI for musculoskeletal, spine, and neck imaging seems promising. Due to common use of metal implants in orthopedic patients, low-field MRI scanners have attracted interest in this field. Some specific designs (e.g. open bores, extremity-specific scanners, and vertical scanners for weight-bearing studies) have facilitated clinical imaging, for example by positioning the limbs in the magnetic field (55). Lee et al. showed an excellent correlation between low- and high-field systems for detection of disk herniation and nerve root compression (56). The application of outpatient neuroimaging by pMRI in neurology and neurosurgery fields has also been studied in recent years. To evaluate the sensitivity of pMRI in detecting multiple sclerosis lesions, Arnold et al. compared 3T MRI with 64 mT pMRI. They showed 94% sensitivity and a strong correlation between the scanners, however, smaller white matter lesions may be missed by pMRI (57). Ventricular volumes in hydrocephalous could be successfully estimated by pMRI compared to conventional MRI and the measurements were strongly correlated. Implanted shunts were similarly affected by magnetic mechanism; However, with reduced interference compared to high-field MRI (58). Interestingly, high-quality cognitive data collection can be achieved through remote neuroimaging and is preferred by patients in a study conducted by Deoni et al. They proposed that pMRI may be an important tool in neuroimaging research (59). Due to the limitation of data regarding pediatric neurodevelopment in lower and middle-income countries, Deoni et al. conducted a study on volumetric measurements and developmental patterns by a 64 mT pMRI in children 6 weeks to 16 years. The authors reported higher success rates with 64 mT pMRI (89%) compared to 3T MRI (75%). Nonetheless, some sequences and analytical software needed to be optimized for pediatric populations. They suggested that implementation of low-field pMRI may provide a more profound understanding of neurodevelopmental patterns among children in low-resource countries that are affected by malnutrition, hydrocephaly, infections, psychosocial challenges, and other environmental factors (60).

## Disadvantages

Some studies have reviewed the clinical challenges of pMRI. Examples include lower quality of DWI sequences on portable scanners and the potential risk of overlooking pathologies; Identifying stroke penumbra and vasculature, i.e. the infarct core and the collateral flow; need for skilled personnel for running the scanner, reading the results, and providing therapy on-site (with the ability to administer tPA) (9, 55). Additionally, the dangers of transferring the scanner through hospital floors; the field shield needed to protect nearby pacemakers, defibrillators, implants, foreign metal bodies, etc.; and possible hygiene issues by moving tools that may serve as a source of spreading pathogens are some of the other potential limitations that can be proposed and further studies are needed to address these issues.

## Discussion

The studies and technologies described in this review demonstrate that low-field pMRI can be successfully integrated into the clinical workflow to provide timely, accurate, and accessible information for the detection of major acute neurological pathologies such as stroke, hemorrhage, and lesions with mass effect. It is particularly useful in settings where cost, space, or scan-time cause limitations; such as the emergency department, critical care setting, mobile stroke units, and resource-limited environments. By serial measurements, pMRI can potentially enhance insight into the dynamic profile and time course of neurological pathologies and hold promise for preventing the progression of neurological complications. However, further studies aimed at establishing the real added value of POC MRI compared to portable head CT scan and the concordance between POC MRI and conventional MRI in a larger variety of clinical settings, optimizing and improving acquisition and post-processed images, addition of newer sequences, and the ability to perform scans with contrast agents to detect subtle neurological abnormalities are needed (22). Moreover, further work is still needed to fully determine the accuracy of POC MRI, clinical indications and utility, and patient selection.

## Author contributions

AS: Writing—original draft, Writing—review and editing. DC: Writing—review and editing. EK: Writing—review and editing. VY: Supervision, Writing—review and editing.
